# A Potential Role for the Interaction of *Wolbachia* Surface Proteins with the *Brugia malayi* Glycolytic Enzymes and Cytoskeleton in Maintenance of Endosymbiosis

**DOI:** 10.1371/journal.pntd.0002151

**Published:** 2013-04-04

**Authors:** Elena Melnikow, Shulin Xu, Jing Liu, Aaron J. Bell, Elodie Ghedin, Thomas R. Unnasch, Sara Lustigman

**Affiliations:** 1 Molecular Parasitology, Lindsley F. Kimball Research Institute, New York Blood Center, New York, New York, United States of America; 2 Department of Global Health, University of South Florida, Tampa, Florida, United States of America; 3 Electron Microscopy, Lindsley F. Kimball Research Institute, New York Blood Center, New York, New York, United States of America; 4 Department of Computational & Systems Biology, Center for Vaccine Research, University of Pittsburgh School of Medicine, Pittsburgh, Pennsylvania, United States of America; Monash University, Australia

## Abstract

The human filarial parasite *Brugia malayi* harbors an endosymbiotic bacterium of the genus *Wolbachia*. The *Wolbachia* represent an attractive target for the control of filarial induced disease as elimination of the bacteria affects molting, reproduction and survival of the worms. The molecular basis for the symbiotic relationship between *Wolbachia* and their filarial hosts has yet to be elucidated. To identify proteins involved in this process, we focused on the *Wolbachia* surface proteins (WSPs), which are known to be involved in bacteria-host interactions in other bacterial systems. Two WSP-like proteins (*w*Bm0152 and *w*Bm0432) were localized to various host tissues of the *B. malayi* female adult worms and are present in the excretory/secretory products of the worms. We provide evidence that both of these proteins bind specifically to *B. malayi* crude protein extracts and to individual filarial proteins to create functional complexes. The *w*Bm0432 interacts with several key enzymes involved in the host glycolytic pathway, including aldolase and enolase. The *w*Bm0152 interacts with the host cytoskeletal proteins actin and tubulin. We also show these interactions *in vitro* and have verified that *w*Bm0432 and *B. malayi* aldolase, as well as *w*Bm0152 and *B. malayi* actin, co-localize to the vacuole surrounding *Wolbachia*. We propose that both WSP protein complexes interact with each other via the aldolase-actin link and/or via the possible interaction between the host's enolase and the cytoskeleton, and play a role in *Wolbachia* distribution during worm growth and embryogenesis.

## Author Summary

The human filarial parasite *Brugia malayi* harbors a *Wolbachia* endosymbiotic bacterium that is required for normal reproduction and development. However, the molecular basis of how this essential endosymbiotic relationship is maintained is not understood. As a first step in trying to understand the molecular interactions that might be essential in this process, we focused on the *Wolbachia* surface proteins (WSPs), which are known to be involved in bacteria-host interactions in other systems. Our aim was to determine whether there are any functional interactions between some of these WSPs and the proteins produced by the host parasite cells. We found that two of the WSP family members specifically interact with proteins produced by the host. *Wolbachia w*Bm0432 interacted with several key enzymes involved in the host glycolytic pathway, the primary energy-producing pathway in the cell. *Wolbachia w*Bm0152 interacted with the host cytoskeleton. These findings suggest that WSP family proteins might play important roles in both optimization of the energy production pathway in *B. malayi* as well as in anchoring the endosymbiont to the host's cytoskeleton.

## Introduction

Nematodes are responsible for the most common parasitic infections of humans. In particular, the tissue-dwelling filarial nematodes—including *Onchocerca volvulus*, *Loa Loa*, *Wuchereria bancrofti*, *Brugia timori* and *B. malayi* (*Bm*)—cause the most severe pathologies associated with these infections, including blindness, extensive skin lesions (in long-standing disease) and elephantiasis [1]–[3]. *O. volvulus*, the causative agent of onchocerciasis, affects nearly 37 million people in 34 countries and is most abundant in Africa, with small foci in Southern and Central America [3]. Approximately 120 million individuals are infected with the causative agents of lymphatic filaria *W. bancrofti* and *B. malayi*, and 40 million exhibit clinical manifestations of disease [4], [5]. The present control programs are based on the mass administration of a small arsenal of microfilaricidal drugs, and thus are vulnerable to possible failure due to the potential development of drug resistance [5]–[9]. Additional research is critically needed to support the discovery of novel drug targets, and thus expand the arsenal of agents targeting the adult worm for the ultimate elimination of these infections [8].

Most filarial parasite species carry a *Wolbachia* endosymbiont, a member of a genus of intracellular bacteria commonly found in arthropods [10], [11]. In insects, *Wolbachia* are primarily reproductive parasites [12]–[14]. Therefore, much of the research on *Wolbachia* endosymbiosis in arthropods has focused on the phenotypic changes caused by infection with the endobacterium, as well as the potential practical applications of the phenotypic alterations, which include cytoplasmic incompatibility, feminization, reduction in host longevity [10] and resistance to viruses and parasites [15]. In filarial nematodes, *Wolbachia* appear to have evolved toward a mutualistic interaction. Spurred by the availability of the genome data from both *B. malayi* [16] and its *Wolbachia* endosymbiont (*w*Bm) [17], research initially focused on pathways that appeared to be defective in one organism and compensated for by genes expressed in the symbiotic partner. Such comparative research suggested that the intact biosynthetic pathways for haem, nucleotides, riboflavin, and FAD comprise the contributions potentially made by the bacteria to the development and survival of the filarial nematodes [1], [18], [19]. Conversely, the *w*Bm genome lacks the complete biochemical pathways for *de novo* synthesis of biotin, coenzyme A, NAD, ubiquinone and folate. Therefore, the filarial worms might provide these and other molecules required for bacterial growth [16], [17].

The co-dependency between *Wolbachia* and the filarial worms was demonstrated by examining the worms after elimination of *Wolbachia* by treatment with antibiotics such as tetracycline, doxycycline or rifamycin [3], [20], [21]. Antibiotic treatments in multiple *in vitro* and *in vivo* studies, including several clinical trials in humans, were shown to induce an apoptotic response in treated parasites [22] leading to strong anti-filarial effects, confirming the essential role of *Wolbachia* in worm survival and reproduction [23]–[30]. For instance, in the Onchocercidae, antibiotic treatment induced retarded larval growth [31], embryostasis in female worms [32], and even death of the adult filarial worms [3], [33]. As the survival and reproduction of the filarial host is dependent on the presence of *Wolbachia* and its interactions with the endosymbiont, this essential interaction has been the subject of intensive studies to identify the Achilles' heel of the symbiotic relationship and thus novel putative chemotherapeutic targets for the treatment of filarial infections [3], [18], [34], [35].

To date, however, little is known about the underlying molecular basis for the *B. malayi* - *Wolbachia* co-dependency. In arthropods, a *Wolbachia* surface protein (WSP) was thought to be a key player for the establishment and persistence of symbiosis, but little is known about the role of this protein or its possible interacting partners in arthropods [36]. The *Wolbachia* surface proteins in filaria were hypothesized to interact with host proteins in the formation of functional complexes necessary for worm survival [20], [36]. The *B. malayi* endosymbiont *Wolbachia* has seven outer membrane proteins (OMPs) and WSPs [17]. These proteins are highly conserved in *Wolbachia* from filarial nematodes and have a heterogeneous pattern of amino acid diversity characteristic of other OMPs known to be involved in bacteria-host interactions in other systems [36]–[39]. Moreover, analysis of the *B. malayi* secretome established that a number of *Wolbachia* OMPs were secreted or released by the worm [40]. In a recent study, an interacting pair of proteins comprised of a WSP-like protein (*w*Bm0284) and a *B. malayi* protein expressed in the cytoplasm of the worms (Bm1_46455, accession# EDP30508.1) was identified [41]. The co-localization of both proteins in similar locations within *Wolbachia* as well as in the worm's tissues, cuticle and nuclei within embryos provided indirect evidence that this specific interaction might have functional importance for the filarial nematode and *Wolbachia* symbiosis [41].

In this study, we focused on two other members of the OMP/WSP protein family of *Wolbachia*, *w*Bm0432 and *w*Bm0152. First, we demonstrated that both *w*Bm surface proteins bind specifically to *B. malayi* crude protein extracts. Second, using *in situ* cross-linking methodology of metabolically labeled worms, we established that *w*Bm0432 interacts *in vivo* with several key glycolytic enzymes (GEs): fructose-bisphosphate aldolase, triosephosphate isomerase, L-lactate dehydrogenase, enolase, glyceraldehyde-3-phosphate dehydrogenase (G3PD), and phosphoglycerate kinase. Notably, *Wolbachia* lacks two glycolytic enzymes (6-phosphofructokinase and pyruvate kinase), and consequently its glycolytic pathway is thought to be defective and replaced by gluconeogenic enzymes [17], [18]. Accordingly, the energy source utilized by *Wolbachia* will depend on products produced by the *B. malayi* glycolytic pathway, such as pyruvate. Moreover, we show that *w*Bm0152 interacts *in vivo* with the host cytoskeletal proteins. Finally, we confirmed these interactions *in vitro* and verified that *w*Bm0432 and *B. malayi* aldolase, as well as the proteins from the second functional complex *w*Bm0152 and *B. malayi* actin, co-localize to the vacuole surrounding *Wolbachia* within the hypodermal cord in female *B. malayi* worms. We further provide evidence to support the theory that these two complexes—*w*Bm0152/*Bm*-actin and *w*Bm0432/GEs—might be connected to each other via the *B. malayi* aldolase-actin linkage, and/or the possible interaction between the host's enolase and cytoskeletal proteins. The results of this study provide a novel molecular perspective on some of the molecular complexes that support the endosymbiotic relationship between *B. malayi* and *Wolbachia*.

## Materials and Methods

### Ethics statement

All animal studies were carried out in compliance with the guidelines from the Institutional Animal Care and Use Committee (IACUC) of the New York Blood Center and in accordance with the recommendations in the Guide for the Care and Use of Laboratory Animals from the National Institutes of Health. The animal protocol (#224) was approved by the IACUC of the New York Blood Center, New York, NY.

### Cloning, expression and purification of the *Wolbachia* surface proteins and *B. malayi* aldolase

The cDNA corresponding to the *Wolbachia* surface protein genes *w*Bm0152 and *w*Bm0432, as well as *B. malayi* aldolase, were amplified by PCR from female *B. malayi* random-primed cDNA using gene-specific primer sets (Table 1). Cloning, expression and purification of corresponding recombinant His tagged proteins in *E. coli* was performed according to a previously reported procedure [41]. The ∼28 kDa His-*w*Bm0432 and 48 kDa His-*Bm*-aldolase fusion protein was purified under denaturing conditions in 6 M urea using His Bind Columns (Novagen), according to the manufacturer's instructions and then dialyzed using 50 mM Tris-HCl, 18 mM NaCl, 1 mM EDTA, pH 7.6. The soluble 18 kDa His-*w*Bm0152 fusion protein was purified using His•Bind Columns (Novagen), according to the manufacturer's instructions and then dialyzed with PBS. The purified recombinant proteins were analyzed by SDS-PAGE. The protein concentration was determined using NanoDrop 2000 (Thermo Scientific).

**Table 1:** Sequences of gene specific primers used for cloning.

Gene name	ID	Primers
Outer membrane protein	*w*Bm0152	5′-CACCGATACATTACTTGATGTAATGGAAG-3′
		5′-CTATTTTTTCATTCCAGAAAATGA-3′
Outer surface protein Wsp	*w*Bm0432	5′-CACCTCTGCTTTTTCAGATCCTGTTGGT-3′
		5′-TTAGAAATTAAACGCTATTCCAGCT-3′
Fructose-bisphosphate aldolase	Bm1_15350	5′-CACCATGACTTCCTACTCTCAGTT-3′
		5′-ATCTAGTACGCATGATTAGCAACA-3′

### Binding of WSPs to *B. malayi* crude protein extracts using an ELISA-based binding assay

*B. malayi* adult female worms (from 120 days post infection of Mongolian jirds) were obtained from the NIAID/NIH Filariasis Research Reagent Repository Center (FR3; Athens, GA; www.filariasiscenter.org). Soluble phosphate buffered saline (PBS) crude protein extracts of *B. malayi* female worms were prepared as described previously [42] using Protease Inhibitor Cocktail (Roche, Mannheim, Germany). Soluble crude protein extract from adult *A. viteae* female worms was prepared in PBS (pH 7.4) containing N-alpha -p-tosyl-L-lysine chloromethyl ketone (50 µg/ml), N-tosyl-L-phenylalanine chloromethyl ketone (50 µg/ml), and phenylmethylsulfonyl fluoride (1 mM) using a glass hand held homogenizer. The *A. viteae* extract was a gift from Drs. William Harnett and Katrina Houston from the University of Strathclyde, Glasgow, Scotland.

A 96-well polystyrene plate (Corning Inc., Corning, NY, USA) was coated with parasite crude protein extract (10 µg/ml) in 0.1 ml of PBS (pH 7.2) overnight at 4°C. The wells were then washed 5 times with PBS-T (PBS plus 0.05% Tween 20) and blocked with 3% BSA in PBS-T for 1 h at room temperature to prevent nonspecific binding. After an additional washing step (5 times with PBS-T) His-*w*Bm0152 or His-*w*Bm0432 recombinant fusion proteins were added to duplicate wells at different concentrations (1–10 µg/ml) in binding buffer, and incubated for 2 h at room temperature. 3% BSA in PBS-T was used as a control for non-specific binding of the detecting antibodies to the parasite extracts. Wells were washed three times with PBS-T and the bound His-tagged protein was detected by probing with HRP conjugated mouse anti-His monoclonal antibody (Genscript) followed by development with a tetramethyl benzidine substrate (Thermo Scientific), and reading the absorbance at 450 nm using SpectraMAX190 (Molecular Devices). The ELISA-based assay was repeated 3 times using crude protein extracts prepared from different batches of *B. malayi* worms and one batch of *A. viteae* extract. The BSA background values were subtracted from the WSPs test wells absorbance. The absorbance in the control wells was consistently below 0.08.

### Production of antibodies against *w*Bm0432 and *w*Bm0152 recombinant proteins

A group of five female BALB/c mice was immunized subcutaneously with 30 µg of recombinant His-*w*Bm0152 or His-*w*Bm0432 formulated in Sigma Adjuvant System as recommended by the manufacturer (Sigma-Aldrich, St. Louis, MO, USA) using an approved IACUC protocol (#224). Boost immunizations were given on days 14 and 28 post-immunization. Blood was collected pre-immunization and on day 14 after the second boost. Pooled serum was analyzed by Western blot. The corresponding bands of the recombinant His-*w*Bm0152 and His-*w*Bm0432 proteins as well as their corresponding native proteins in the *B. malayi* crude protein extract were detected using the antigen-specific antibodies, whereas no bands were recognized when pre-immunization serum was used (data not shown).

### Affinity purification of putative *Bm*–*w*Bm protein complexes

We adapted a method used routinely for protein-protein interaction studies in mammalian cells –*in vitro* metabolic labeling with L-Photo-Leucine and L-Photo-Methionine amino acids, followed by photo-activated *in vivo* cross linking and purification of protein complexes for analysis [42]. Thermo Scientific L-Photo-Leucine and L-Photo-Methionine are analogs of L-Leucine and L-Methionine amino acids that have activatable diazirine side chains capable of chemical crosslinking to adjacent molecules when exposed to ultraviolet light. When used in combination with specially formulated limiting cell media that is devoid of leucine and methionine, the photo-activatable derivatives are treated like naturally occurring amino acids by the protein synthesis machinery. As a result, they can be substituted for leucine or methionine in the primary sequence of proteins. When exposed to UV light the diazirine rings become reactive intermediates that form covalent bonds with nearby protein side chains and backbones. Naturally associating binding partners are then instantly trapped. Briefly, 300 adult *B. malayi* female worms were incubated overnight in Dulbecco's Modified Eagle's Limiting Medium (DMEM-LM) (Thermo Scientific) containing 2 mM L-Photo-Methionine and 4 mM L-Photo-Leucine (Thermo Scientific) without serum. The next morning the media containing the photo-amino acids was removed from the worms and after washing twice with PBS, the worms were covered with a minimal layer of cold PBS and exposed to UV light (2×15 watt bulbs, emission at 350 nm; F15T8/350BLS/ECO) from a 4 cm distance using the XX-15S Shortwave UV Bench Lamp (UVP, Upland, CA). Under these conditions, the photo-reactive amino acid half-life was determined to be 4 min. We irradiated the worms for 20 min; 5 times the half-life, as recommended by the manufacture. A soluble crude protein extract of the labeled worms, including various naturally associating cross-linked binding partners, was prepared in PBS as described above. The protein concentration was determined using NanoDrop 2000 (Thermo Scientific).

To affinity purify putative *Bm*–*w*Bm protein complexes associated specifically with *w*Bm0152 or *w*Bm0432, affinity columns containing IgG raised against *w*Bm0152 or *w*Bm0432 and immobilized to Protein A/G Plus Agarose were prepared using the Pierce Crosslink Immunoprecipitation Kit (Thermo Scientific) according to the instruction of the manufacturer. The PBS soluble crude protein extract prepared from the metabolically-labeled *B. malayi* worms containing the UV induced cross-linking of interacting proteins was first precleared with normal mouse IgG immobilized to Protein A/G. Precleared aliquots of the extract were then loaded onto the *w*Bm0152 or *w*Bm0432 immunoaffinity columns and allowed to incubate overnight at 4°C. After extensive washes of the columns, the bound material was eluted using elution buffer, neutralized with TRIS-HCl, and then analyzed by Western blotting. The eluted material (6 µg per lane) was loaded on a 12% SDS-Tris-glycine gel (Bio-Rad), and the corresponding nitrocellulose membrane strips were then washed, blocked with 1× Casein, and probed with primary mouse anti-*w*Bm0152 or mouse anti-*w*Bm0432 antibodies. Binding was detected using goat anti-mouse secondary antibodies conjugated to horseradish peroxidase (KPL), and a chemiluminescent substrate (SuperSignal).

### MS-based protein identification of the *w*Bm0152- or the *w*Bm0432-specifically bound *B. malayi* material

MS-based protein identification of the *w*Bm0152- or *w*Bm0432-specific bound samples containing putative UV induced cross-linked interacting proteins was initiated by filter-aided sample preparation (FASP), as previously described (Protein Discovery, Knoxville, TN) [43]. Tryptic peptides resulting from the preparation were analyzed by liquid chromatography mass spectrometry (LC-MS). Briefly, chromatography was performed using a Nano-LC Ultra 2D+ (Eksigent, Dublin, CA) equipped with a Proteopep 2 IntegraFrit trapping column (100 µm i.d.×2.5 cm; C18, 5 µm, 300 ???) and a Proteopep 2 IntegraFrit analytical column (75 µm i.d.×10 cm; C18, 5 µm, 300 ???, New Objective, Woburn, MA). Samples were loaded onto the trap column at 2 µl/min (Solvent A) for 12 minutes, after which a valve was switched to include the analytical column. Peptides were then eluted with a gradient (300 nl/min) of 2% B to 80% B over 80 minutes (Solvent A: 97.5% H_2_O, 2% acetonitrile, 0.5% formic acid, Solvent B: 1.5% H_2_O, 98% acetonitrile, 0.5% formic acid). Nano-LC effluent was analyzed on-line by positive-ion micro-electrospray with a linear ion trap (LTQ XL) or LTQ OrbiTrap XL (Thermo Fisher Corp., Bremen, Germany) operated in ‘top-5 data-dependent’ acquisition mode. Resulting data were searched against a custom built database with MASCOT (Matrix Science, Boston, MA). Identified peptides and proteins were validated and visualized with Scaffold 3.6 (Proteome Software, Portland, OR) at a 2% false positive rate.

### Overlay assays for protein-protein interaction analyses

#### Interaction of *w*Bm0432 with rabbit Glycolytic enzymes (GEs)

The overlay assays were performed as previously described [44]. In brief, rabbit GEs (Sigma) (Table 2), 3 µg per each lane, were run on SDS-PAGE and transferred to nitrocellulose membranes. The blots were blocked overnight with an overlay assay buffer (50 mM HEPES, pH 7.3, 3 mM EGTA, 3 mM CaCl2, 3 mM MgCl2, 80 mM KC1) containing 0.1 mM DTT and 5% (w/v) skim milk at 4°C, and then incubated with the same buffer containing 3 µg/ml purified His-*w*Bm0432 for 3 h at room temperature. The membranes were washed several times with Tris buffered saline (20 mM Tris-HCl, pH 7.5, 150 mM NaCl) containing 0.05% Tween 20 before being reacted with HRP conjugated mouse anti-His monoclonal antibody (Genscript) overnight at 4°C. The membranes were developed using an enhanced chemiluminescence kit (Pierce) as described by the manufacturer.

**Table 2:** GEs are conserved between *O. cuniculus* and *B. malayi*.

Protein name	*B. malayi* (ID)	*B. malayi* (Accession#)^a^	*O. cuniculus* (Accession#)	Sequence Identity
Fructose-bisphosphate aldolase	Bm1_15350	EDP36623.1	NP_001075707.1	69%
Triosephosphate isomerase	Bm1_29130	EDP33878.1	P00939.1	62%
L-lactate dehydrogenase	Bm1_43730	EDP30958.1	NP_001182636.1	60%
Enolase	Bm1_24115	EDP34873.1	XP_002716189.1	72%
Glyceraldehyde-3-phosphate dehydrogenase	Bm1_41940	EDP31080.1/EDP31079.1	XP_002708267.1	69%
Phosphoglycerate kinase	Bm1_01925	EDP39298.1	XP_002709046.1	73%

a BLAST searches were used to identify *O. cuniculus* GEs orthologs in *B. malayi*.

#### Interaction of *w*Bm0432 with *B. malayi* aldolase

*Bm*-aldolase (3 µg per lane) was separated by SDS-PAGE and transferred to nitrocellulose membrane. The membrane blot was blocked overnight with overlay assay buffer at 4°C and then incubated with the same buffer containing 2.5 µg/ml of purified His-*w*Bm0432 for 3 h at RT. To determine the *K_d_* values for the *w*Bm0432 interaction with *B. malayi* aldolase, individual strips containing aldolase were incubated with 1 µg/ml, 2 µg/ml, 5 µg/ml or 10 µg/ml purified His-*w*Bm0432 in overlay assay buffer for 3 h at room temperature. The membranes were washed and developed as described above. The signals were quantified using GeneSnap (Syngene) software.

#### Interaction between *w*Bm0152 and actin and between *Bm-*aldolase and actin

*w*Bm0152 or *Bm-*aldolase, 3 µg per lane, was separated by SDS-PAGE and transferred to nitrocellulose membranes. The individual strips were blocked overnight with overlay assay buffer at 4°C and then incubated with the same buffer containing 2.5 µg/ml purified bovine actin (sequence identity with *Bm*-actin is 98%) for 3 h at RT. The membranes were washed as described above and reacted first with Goat anti-actin (Santa Cruz) followed by the HRP-conjugated polyclonal anti-goat antibodies (Santa Cruz) for 3 h. After the washings, the membranes were developed as described above.

### ELISA-based protein binding assay to test the interaction between recombinant *w*Bm0152 and actin

To verify the putative protein-protein interaction between the recombinant His-*w*Bm0152 and actin we used the ELISA-based assay. The 96-well polystyrene plates (Corning Inc.) were coated with the purified recombinant His-*w*Bm0152 protein at 0.2 µg/ml or 1 µg/ml as described above. The reactant bovine actin protein (Sigma) was then added in duplicates at 1 µg/ml, 5 µg/ml or 20 µg/ml. The bound actin was detected using rabbit anti-actin polyclonal antibody (Genscript) followed by HRP-conjugated goat anti-rabbit IgG (KPL). HRP was detected as described above.

### Localization of *w*Bm0152 and actin and *w*Bm0432 and *Bm*-aldolase in *B. malayi* worms by immunoelectron microscopy

*B. malayi* female worms were fixed in a mixture of 4% paraformaldehyde and 0.25% glutaraldehyde in 0.1 M sodium cacodylate buffer (pH 7.4) containing 1% sucrose for 60 min at room temperature and processed for immunoelectron microscopy as described previously [45]. Thin sections of embedded worms were blocked and probed with rabbit antibodies raised against recombinant His-*w*Bm0432 (1∶ 5 dilution) and mouse anti-*Bm*-aldolase (1∶2 dilution) antibodies followed by 15 nm or 10 nm gold labeled goat anti-rabbit IgG (H+L) or 18 nm gold labeled goat anti-mouse IgG (H+L) (Jackson ImmunoResearch Laboratories, Inc., USA), respectively. Similarly, thin sections of embedded worms were blocked and probed with mouse antibodies raised against recombinant His-*w*Bm0152 (1∶2 dilution) and rabbit anti-actin antibodies (1∶20 dilution) followed by 15 nm or 18 nm gold labeled goat anti-mouse IgG (H+L) or 15 nm gold labeled goat anti-rabbit IgG (H+L) (Jackson ImmunoResearch Laboratories, Inc., USA), respectively. Pre-immunization serum was used as the control. No signals were detected in control experiments utilizing pre-immunization sera (data not shown).

In addition, worms were processed for transmission immunoelectron microscopy as described above with the exception of sectioned material being post stained with 1% tannic acid, 2% osmium tetroxide, saturated ethanolic uranyl acetate and Reynolds lead citrate. Regular epon embedding was also performed on the same sample in order to compare the effects of the two different fixation protocols on the morphology of the *B. malayi* host vacuole surrounding *Wolbachia*. Epon embedded processing consisted of fixing the worms in modified Karnovsky's fixative consisting of 2.5% glutaraldehyde and 2% paraformaldehyde in 0.1 M sodium cacodylate buffer, pH 7.4, containing 1% sucrose for 60 minutes at room temperature. Worms were then washed 3×10 min in 0.1 M sodium cacodylate buffer and post fixed with 2% osmium tetroxide for 60 min. Following additional buffer washes, worms were dehydrated through an ethanol series and immersed in propylene oxide for 2×10 min before being embedded in Epon resin. Ultrathin sections were cut using an RMC MTX ultramicrotome with a Diatome diamond knife followed by post staining of the grids with saturated ethanolic uranyl acetate and Reynolds lead citrate. Samples were imaged on a FEI Tecnai 12 spirit TEM operated at 80 kV.

## Results

### Recombinant *w*Bm0152 and *w*Bm0432 WSP-like proteins bind specifically to *B. malayi* protein extracts

**Figure 1:**
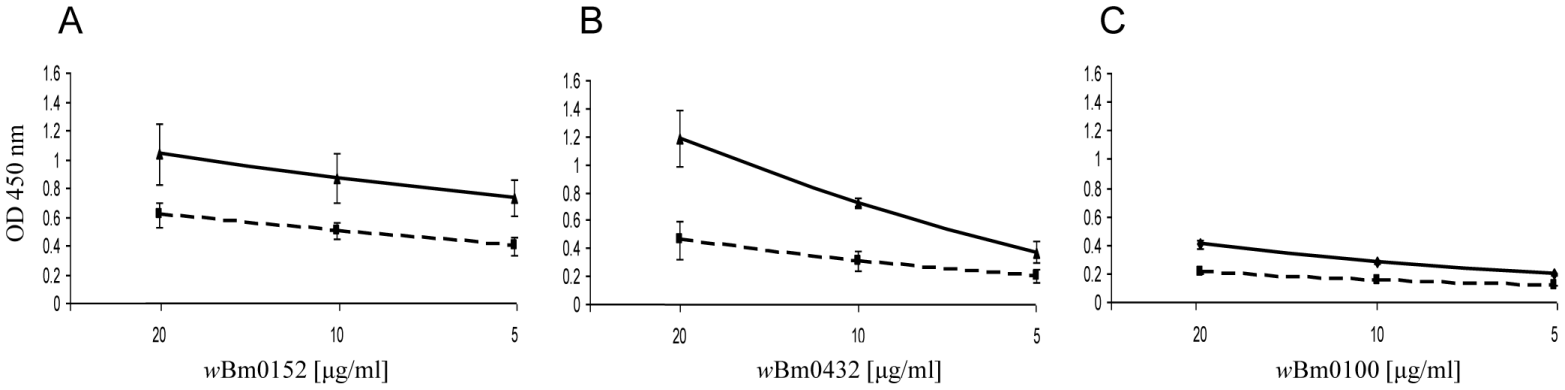
ELISA-based assay to test for possible interaction between *Wolbachia* WSP-like proteins and crude protein extracts of parasitic filarial nematodes. Plates were coated with 10 µg/ml of crude protein extract prepared from *B. malayi* (which contains the *w*Bm; solid line) or *A. viteae* (which lacks the endosymbiont; dashed line) and were then exposed to varying concentrations of purified recombinant His-tagged *w*Bm0152 (A), *w*Bm0432 (B), or *w*Bm0100 (C). The binding was detected using HRP-conjugated anti-His antibodies. OD: optical density. Data represent the mean absorbance at 450 nm ± S.D. of three independent experiments. The binding of recombinant WSP protein to *B. malayi* crude extracts was compared with its binding to *A. viteae* crude extract by a Student's two-tailed t test (*P*<0.05).

**Figure 2:**
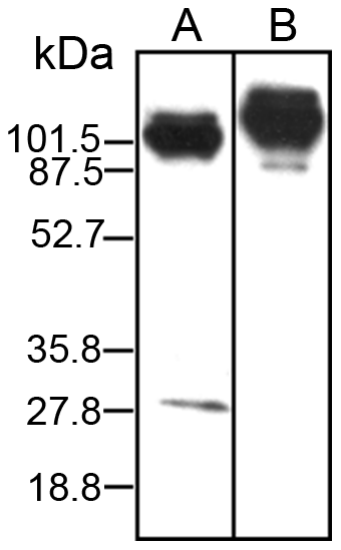
Detection of *Bm*-*w*Bm protein complexes within crude cross-linked metabolically labeled worm extract using anti-*w*Bm0432 or anti-*w*Bm0152 antibodies. L-Photo-Leucine and L-Photo-Methionine metabolically labeled *B. malayi* worms were cross-linked for 20 minutes using UV followed by soluble protein crude extract preparation. The soluble extract was passed through an affinity column containing Protein G cross-linked to normal mouse IgG followed by affinity columns containing anti-*w*Bm0432 or anti-*w*Bm0152 antibodies cross-linked to Protein G. The corresponding protein eluates (6 µg/Lane) were run on a SDS-PAGE prior to Western blotting with (A) mouse anti-*w*Bm0432 specific antibodies (bands: ∼110 & 28 kDa) or (B) mouse anti-*w*Bm0152 specific antibodies (bands: ∼90 kDa and ∼120 kDa).

To evaluate the possible interaction between the *Wolbachia* surface proteins and *B. malayi* proteins, we utilized an *in vitro* ELISA-based assay [41] using recombinant His-tagged WSPs and *B. malayi* crude protein extract. The worm's components contained in the *B. malayi* soluble crude protein extract were immobilized on the ELISA plates and then incubated with varying concentrations of the recombinant His-tagged WSP proteins of *w*Bm0100, *w*Bm0152 or *w*Bm0432. The crude protein extract of *Acanthocheilonema viteae*, a filarial nematode that is free of *Wolbachia*, was used as a control for possible non-specific binding [12], [31], [41], [46]. As shown in Figures 1A and 1B, 2 out of the 3 WSP proteins, *w*Bm0152 and *w*Bm0432, bound specifically (*P*<0.05) in a dose-dependent manner to the *B. malayi* crude protein extract, whereas these *Wolbachia* proteins exhibited minimal binding capacity to the *A. viteae* crude protein extract using similar assay conditions. Based on the data presented later, it is possible that the minimal binding to the *A. viteae* crude protein extract observed is due to cross-reactivity with the glycolytic enzymes or the actin/tubulin proteins in *A. viteae* that are presumed to be highly similar to those of *B. malayi*. Notably, *w*Bm0100 did not bind to the *B. malayi* crude protein extract (Fig. 1C). These results indirectly established the presence of putative binding partners within the *B. malayi* crude protein extract that bind more specifically with the *Wolbachia* surface proteins *w*Bm0152 and *w*Bm0432.

### Detection of *B. malayi* – *Wolbachia* WSP protein complexes within metabolically-labeled adult female worm crude protein extracts containing UV induced cross-linked interacting proteins

To identify the possible *B. malayi* interacting partners of *w*Bm0152 and *w*Bm0432 *in vivo*, we adapted a method used routinely for protein-protein interaction studies in mammalian cells–*in vitro* metabolic labeling with L-Photo-Leucine and L-Photo-Methionine amino acids, followed by photo-activated *in vivo* cross linking, and immune-purification of protein complexes for analysis [47]. In these experiments, *B. malayi* adult females were metabolically labeled with L-Photo-Leucine and L-Photo-Methionine amino acids, the parasites lysed and the metabolically labeled proteins photo-cross-linked. The crude protein extract prepared from these cross-linked metabolically-labeled *B. malayi* worms was first precleared by passing it over an immunoaffinity column consisting of IgG from a naive mouse immobilized with Protein A/G. The native *Wolbachia*–*B. malayi* complexes were then affinity-purified using IgG from mice immunized with recombinant *w*Bm0152 or *w*Bm0432, again immobilized with Protein A/G. The corresponding eluted fractions from anti-*w*Bm0152 and anti-*w*Bm0432 immunoaffinity columns were then analyzed by Western blot. An antiserum against *w*Bm0432 revealed two discrete bands in the eluted fraction: ∼28 kDa and ∼110 kDa (Figure 2A). The expected molecular weight (MW) of an unassociated native *w*Bm0432 molecule is 26 kDa. Therefore, we concluded that the lower band represented the native unbound *w*Bm0432, and that the higher broad band indicated the presence of some putative *w*Bm0432 – *B. malayi* protein complexes. The anti-*w*Bm0152 antibodies reacted with eluted proteins of ∼90 kDa and ∼120 kDa (Fig. 2B). As the molecular weight of the native protein *w*Bm0152 protein is only 18 kDa, we concluded that the two recognized protein bands correspond to some possible *w*Bm0152 – *B. malayi* protein complexes. Both of the affinity purified fractions containing the putative protein complexes of *Wolbachia* WSP and *B. malayi* proteins were analyzed by mass spectrometry.

### The *w*Bm0432 interacts with the host glycolytic enzymes and *w*Bm0152 interacts with *B. malayi* cytoskeleton proteins

The identity of the proteins contained within the affinity-purified complexes were resolved using liquid chromatography mass spectrometry (LC-MS) [48]. The complexes purified using the anti-*w*Bm0432 affinity column contained 9 peptides corresponding to the sequence of the native *w*Bm0432 protein. In addition, peptides derived from six *B. malayi* proteins involved in the glycolytic pathway were found in the digested affinity-purified complexes: fructose-bisphosphate aldolase, triosephosphate isomerase, enolase, glyceraldehyde-3-phosphate dehydrogenase and phosphoglycerate kinase, and L-lactate dehydrogenase (Table 3). The protein with the most abundant tryptic peptides (five) was fructose-bisphosphate aldolase (Bm1_15350, accession# EDP36623.1) (Table 3). The proteins eluted from the anti-*w*Bm0152 affinity column included actin (Bm1_16810, accession# EDP36330.1) and α- and β- tubulin (Table 3). However, *w*Bm0152 was not included in the list of proteins identified in this analysis. These results suggested that *w*Bm0432 may interact directly or indirectly with six key enzymes involved in the host glycolytic pathway, while *w*Bm0152 may interact with the host cytoskeleton.

**Table 3:** Composition of the *B. malayi* – *Wolbachia* WSP protein complexes identified by LC-MS analyses.

anti-*w*Bm0432^a^			
Protein identified	Accession #	Gene ID	No. peptides detected
Outer surface protein WSP	YP_198262.1	wBm0432	9
Fructose-bisphosphate aldolase **	EDP36623.1	Bm1_15350	5
Triosephosphate isomerase **	EDP33878.1	Bm1_29130	4
Histone H2B	EDP39185.1	Bm1_02510	3
L-lactate dehydrogenase **	EDP30958.1	Bm1_43730	3
Myotactin form B, putative	EDP28983.1	Bm1_53510	3
High mobility group protein 1	EDP34582.1	Bm1_25620	3
Enolase **	EDP34873.1	Bm1_24115	2
Glyceraldehyde-3-phosphate dehydrogenase**	EDP31079.1	Bm1_41940	2
Heat shock 70 kDa protein	EDP30947.1	Bm1_43675	2
Phosphoglycerate kinase **	EDP39298.1	Bm1_01925	2
14-3-3-like protein 2, putative	EDP36044.1	Bm1_18190	2

anti-*w*Bm0152^a^			
Protein identified	Accession #	Gene ID	No. peptides detected
Actin*	EDP36330.1	Bm1_16810	10
Tubulin alpha chain*	EDP33743.1	Bm1_30260	3
Beta-tubulin*	EDP34538.1	Bm1_25780	3
ATP synthase subunit beta	EDP30409.1	Bm1_45960	3
Tubulin alpha chain-mouse*	EDP31997.1	Bm1_38680	2
Elongation factor 1-alpha	EDP37757.1	Bm1_09680	2

a Photo-activated crosslinked adult female *B. malayi* extracts complexes were eluted from affinity columns containing anti-*w*Bm0432 or anti-*w*Bm0152 antibodies bound to protein G.

** : members of the glycolytic enzymes.

* : cytoskeleton complexes.

### The *Wolbachia* surface proteins interact specifically with their *B. malayi* binding partners

**Figure 3:**
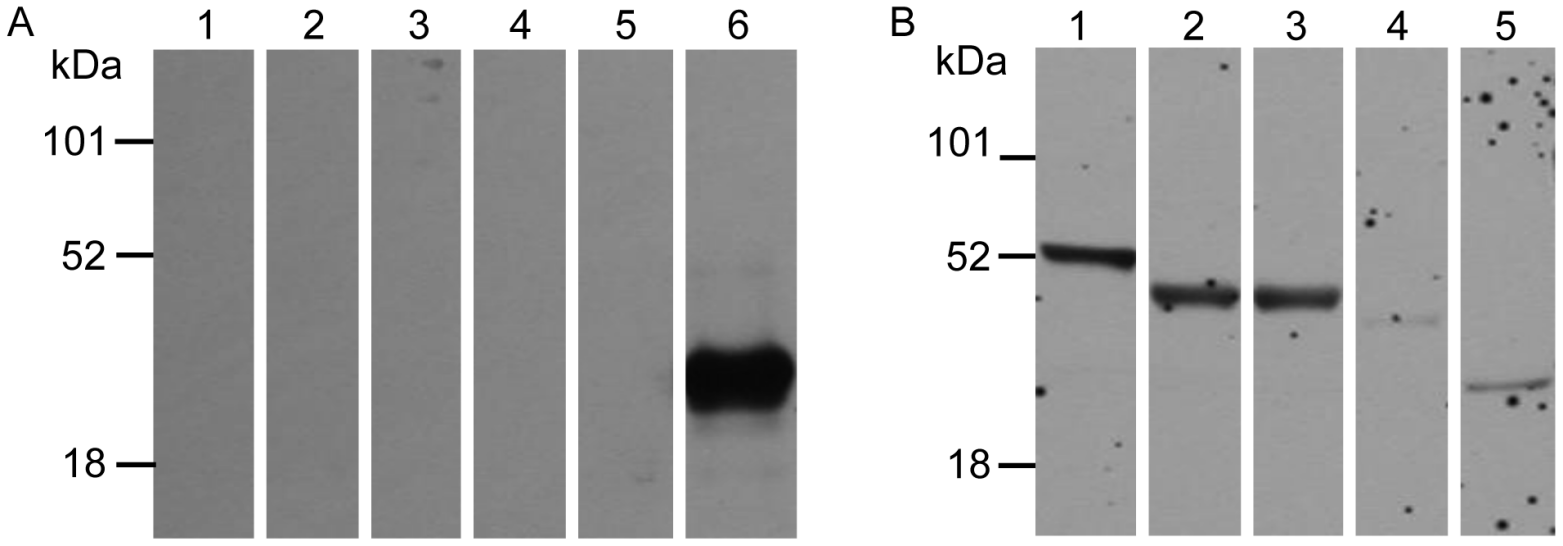
Interaction between *Wolbachia* WSP *w*Bm0432 and glycolytic enzymes *in vitro*. Enolase (Lane 1), aldolase iV (Lane 2), aldolase X (Lane 3), glyceraldehyde-3-phosphate dehydrogenase (Lane 4), triosephosphate isomerase (Lane 5), and recombinant His-tagged *w*Bm0432 (Lane 6), 3 µg each, were blotted onto nitrocellulose membrane and incubated for 3 h with binding buffer (**A**) or with 2 µg/ml of recombinant His-*w*Bm0432 (**B**). Binding was detected using HRP-conjugated mouse anti-His antibodies.

To confirm the interaction between the two *Wolbachia* WSPs and their *B. malayi* partner proteins, we utilized an *in vitro* overlay assay [44]. Initially, we determined which of the GEs interact directly and distinctively with *w*Bm0432 by performing overlay assays using commercially available rabbit glycolytic enzymes, which were immobilized onto nitrocellulose. The sequence identity of *B. malayi* proteins and *Oryctolagus cuniculus* (European rabbit) proteins ranged from 62% to 72% (Table 2). As shown in Figure 3B, *w*Bm0432 binds strongly with enolase (Lane 1) and aldolase (Lanes 2 and 3). The *w*Bm0432 also interacted with triosephosphate isomerase to some extent (Fig. 3B, Lane 5), and had its weakest binding to L-lactate dehydrogenase (Fig. 3B, Lane 4). None of the GEs cross-reacted with the anti-His detecting antibodies (Fig. 3A, Lanes 1–5).

**Figure 4:**
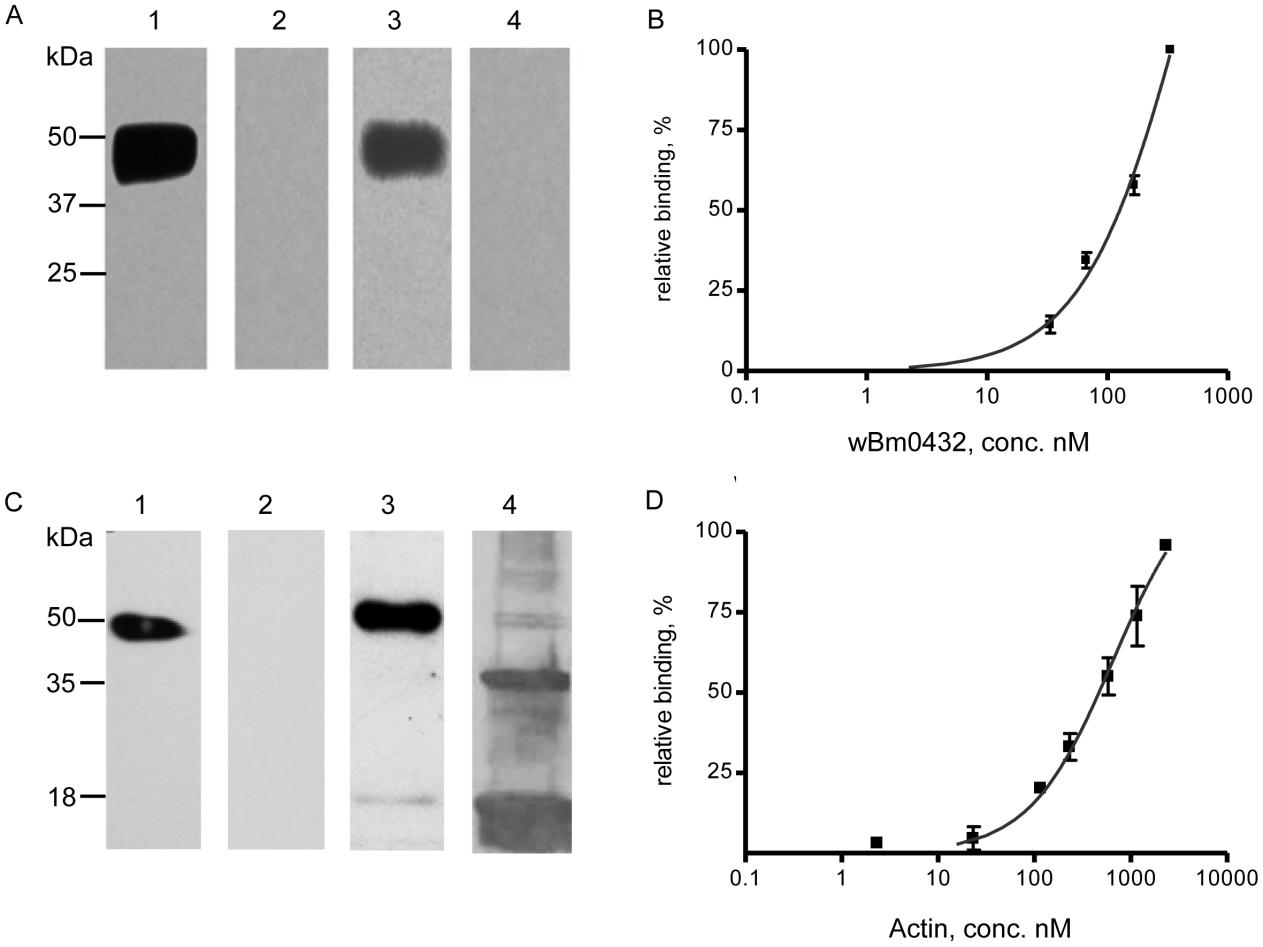
Interaction between *Wolbachia* WSPs and actin or *B. malayi* aldolase *in vitro*. (**A**) Aldolase overlay assay: Recombinant His-tagged *B. malayi* aldolase (3 µg) was blotted onto nitrocellulose membrane and individual strips were incubated for 3 h with: binding buffer (Lanes 1 & 2); 2 µg/ml of recombinant His-*w*Bm0432 (Lane 3); and 2 µg/ml His-*w*Bm0293 (Lane 4), an unrelated *Wolbachia* protein. Binding was detected using the following antibodies: mouse anti-His-*Bm*-aldolase (Lane 1); mouse anti-His-*w*Bm0432 (Lanes 2 & 3), and mouse anti-His-*w*Bm0293 (Lane 4), followed by HRP-conjugated secondary goat anti-mouse antibodies. (**B**) The *K_d_* of *Wolbachia w*Bm0432 and aldolase interaction was determined by an overlay assay using recombinant His-tagged *B. malayi* aldolase (3 µg/Lane) blotted onto nitrocellulose membrane and incubation of the individual strips for 3 h with: 1, 2, 5 or 10 µg/ml of recombinant His-*w*Bm0432. Binding was detected using mouse anti-His-*w*Bm0432. (**C**) Actin overlay assay: Purified bovine actin (3 µg, Lane 1) and recombinant His- *w*Bm0152 (3 µg, Lanes 2–4) were blotted onto nitrocellulose membrane and individual strips were incubated for 3 h with: binding buffer (Lanes 1, 2, 4) or 2.5 µg/ml of bovine actin (Lane 3). Binding was detected using rabbit anti-actin (Lanes 1, 2, 3) or mouse anti-His-*w*Bm0152 (Lane 4) followed by HRP-conjugated goat anti-rabbit or goat anti-mouse antibodies, respectively. (**D**) The *K_d_* of *Wolbachia w*Bm0152 and actin interaction was determined by ELISA-based assay. A plate coated with 20 µg/ml of His-*w*Bm0152 was exposed to varying concentrations of bovine actin. The binding was detected using rabbit Anti-actin polyclonal antibody followed by HRP-conjugated goat anti-rabbit IgG.

To validate that *w*Bm0432 interacts specifically with the filarial host aldolase, we repeated the overlay assay with recombinant *B. malayi* His-tagged aldolase (Bm1_15350) immobilized onto nitrocellulose. As shown in Figure 4A, His-*w*Bm0432 also interacts specifically with His-*Bm*-aldolase (Fig. 4A, Lane 3). Moreover, anti-His-*w*Bm0432 (Fig. 4A, Lane 2) or anti-His-*w*Bm0293, an unrelated *Wolbachia* protein, (data not shown) antibodies did not cross-react with the immobilized aldolase. To determine the experimental dissociation constant (*K_d_*) for the *w*Bm0432 and *B. malayi* aldolase interaction, individual strips of the immobilized recombinant *B. malayi* aldolase were incubated with different concentrations of *w*Bm0432 (Fig. 4B). The calculated *K_d_* value of 0.51±0.2 µM further highlighted the specificity of the interaction between *w*Bm0432 and *B. malayi* aldolase.

As shown in Figure 4C, soluble bovine actin, which is >90% identical to *B. malayi* actin, interacted specifically with the His-*w*Bm0152 protein (Lane 3). The specificity of the anti-actin antibodies (Fig. 4C, Lane 1) was verified by establishing that they did not cross-react with His-*w*Bm0152 (Fig. 4C, Lane 2). Notably, the interaction between actin and *w*Bm0152 is the strongest when the *Wolbachia* protein is polymerized and runs in the gel as a tetramer (∼54 kDa) (Fig. 4C, Lanes 3 and 4). Subsequently, using ELISA-based interaction assays, the experimental dissociation constant (*K_d_*) of *w*Bm0152 and bovine actin was determined to be 0.57±0.03 µM, indicating a high binding affinity [49] (Fig. 4D).

In summary, these *in vitro* binding assays further supported our LC-MS analyses that *w*Bm0432 interacts specifically with *Bm*-aldolase, and that *w*Bm0152 interacts specifically with actin.

### The *Wolbachia* surface proteins *w*Bm0432 and *w*Bm0152 co-localize with their corresponding *B. malayi* binding partners, aldolase and actin, *in vivo*

**Figure 5:**
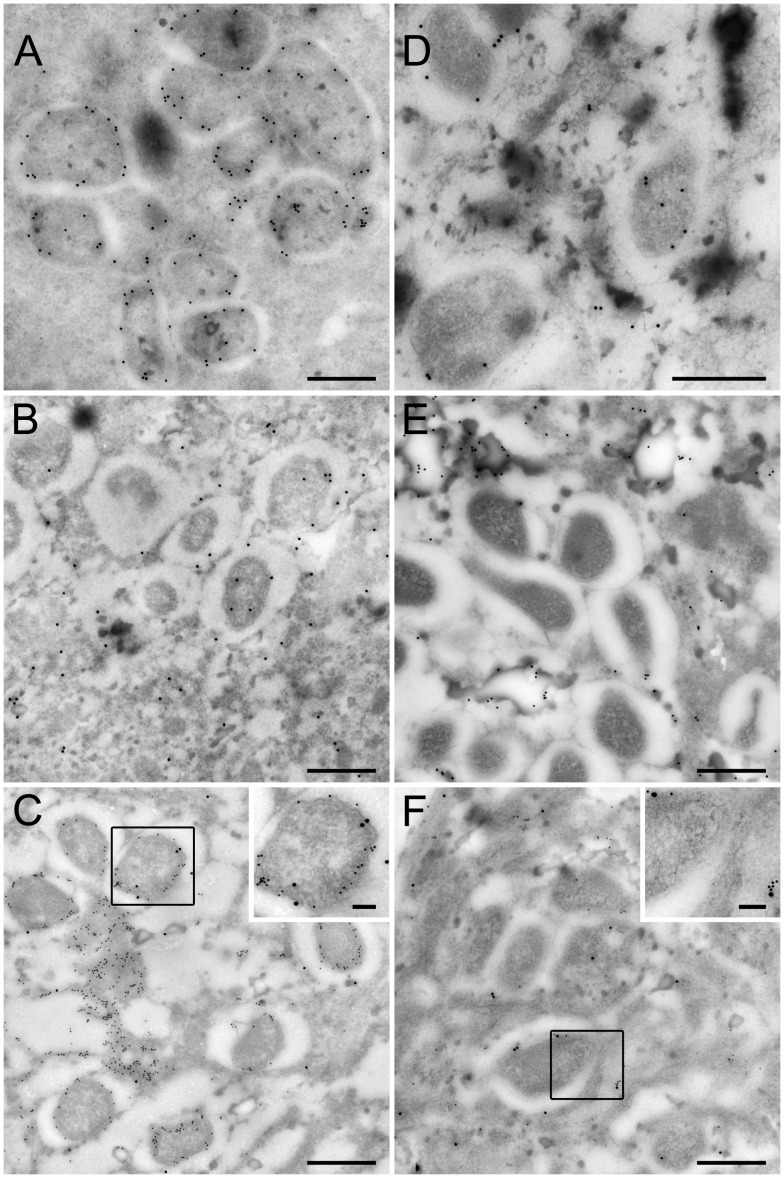
Co-localization of *Wolbachia* WSP protein *w*Bm0432 with *Bm*-aldolase and *w*Bm0152 with *Bm*-actin. Cross sections of the hypodermal cord in female worms were probed with rabbit anti-*w*Bm0432 (A), mouse anti-*Bm*-aldolase (B), or both (C), followed by anti-rabbit gold (15 nm in A and 10 nm in C) or anti-mouse gold (18 nm in B and C). Cross sections of the hypodermal cord in female worms were probed with mouse anti-*w*Bm0152 (D), rabbit anti-actin (E), or both (F) followed by anti-mouse gold (15 nm in D and 18 nm in F) or anti-rabbit gold (15 nm in D and 10 nm in F). Bar: Panels A–F - 500 nm, inserts in Panels C and F - 100 nm. Note the proximity of *w*Bm0432 and aldolase (insert in C) as well as that of the *w*Bm0152 and actin within the *Wolbachia* vacuole (insert in F).

The interaction for each of the two protein complexes, *w*Bm0432-aldolase and *w*Bm0152-actin, was confirmed *in situ* by immunoelectron microscopy using rabbit anti*-w*Bm0432 antiserum and mouse anti-*Bm*-aldolase antiserum, and mouse anti-*w*Bm0152 antiserum and rabbit anti-actin antibodies, respectively. The *w*Bm0432 protein localized to the surface of *Wolbachia* (Fig. 5A and 5C), as previously shown [41], [50]. Immunolocalization of *Bm*-aldolase established that aldolase was also present close to the surface of *Wolbachia* (Fig. 5B and 5C). Subsequent double labeling of similar cross-sections of *B. malayi* adult female worms with both antibodies co-localized the corresponding proteins to the surface of the vacuole that surrounds *Wolbachia* within the cytoplasm of the *B. malayi* host (Fig. 5C).

Additional transmission electron microscopy experiments were performed to examine the structure of the *B. malayi* host vacuole surrounding *Wolbachia* in the hypodermal chord. The appearance of the vacuole was found to be considerably altered in a fixation-dependent manner. Worms processed for immunoelectron microscopy utilizing a less stringent fixation protocol were observed to have a large halo surrounding the bacteria (Fig. S1, Panels A and B). To better examine the host vacuole structure in the immunoelectron microscopy samples, sectioned material was post stained with 1% tannic acid, 2% osmium tetroxide, saturated ethanolic uranyl acetate and Renolds lead citrate in order to stain membranes and microfilament structures associated with the host vacuole. For comparison, worms were processed for structural electron microscopy studies from the same sample but utilizing a more stringent fixation protocol. These samples were found to lack the large halo seen in immunoelectron microscopy preparations (Fig. S1, Panels C and D) and in fact were virtually indistinguishable from the host cytoplasm in some areas.

Closer examination of the vacuole from the samples in Figure S1, Panels A and B revealed that the perceived vacuole boundary was comprised of a dense material that appeared to lack a bilayer membrane that is found in traditional membrane bound vacuoles (black arrow heads, Fig. S2). However, it is possible that a membrane is either masked by the large amount of proteinaceous material present at the boundary, or not well preserved in these samples. In addition, microfilaments were observed to be adjacent to the vacuole boundary (broad white arrows, Fig. S2). Labeling of adult female *B. malayi* worms using mouse anti-*w*Bm0152 antiserum demonstrated that the protein is present on the surface and in the areas surrounding *Wolbachia* (Fig. 5D). Rabbit anti actin antibodies appeared to cross-react with a *B. malayi* actin protein within the tissue surrounding *Wolbachia* (Fig. 5E). Notably, double labeling of similar cross-sections of *B. malayi* adult female worms with both antibodies co-localized the corresponding proteins to the surface of the vacuole that surrounds *Wolbachia* within the cytoplasm of the *B. malayi* host (Fig. 5F). These results verify that the *Wolbachia* WSP proteins, *w*Bm0432 and *w*Bm0152, interact with their corresponding complex partner proteins *Bm*-aldolase and *Bm*-actin within *B. malayi*.

### Aldolase is the putative link between *w*Bm0432/GEs and *w*Bm0152/cytoskeleton protein complexes

**Figure 6:**
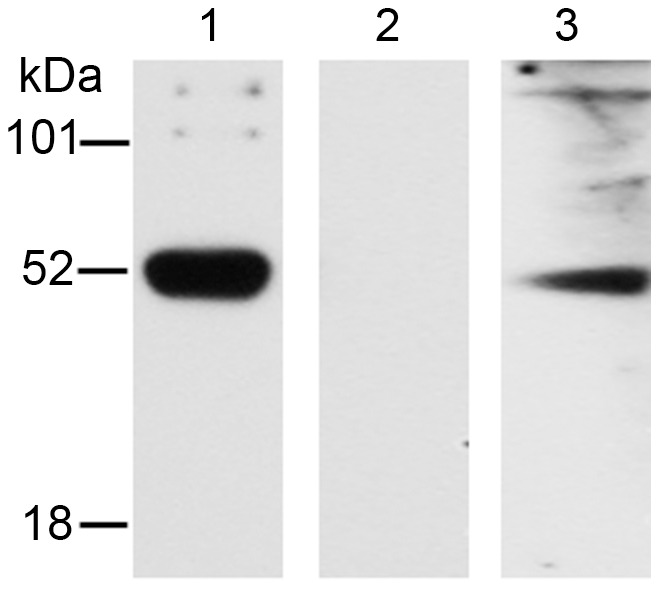
Overlay assay to prove the interaction between *Bm*-aldolase and actin. Actin (Lane 1) or recombinant His-tagged *Bm*-aldolase (3 µg) (Lanes 2 and 3) were blotted onto nitrocellulose membrane and the individual strips were incubated for 3 h with: binding buffer (Lanes 1 and 2) or 2.5 µg/ml of bovine actin (Lane 3). Binding was detected using goat anti-actin followed by HRP-conjugated anti-goat antibodies.

Previous studies have shown that aldolase not only catalyzes a key step in glycolysis but that it is also able to bind to F-actin in cells such as endothelial cells and fibroblasts, as well as in apicomplexan parasites [51]–[53]. Given this role in other organisms, the possible interaction between *B. malayi* aldolase and actin was explored. The overlay assay demonstrated that *B. malayi* aldolase binds specifically to actin (Fig. 6, Lane 3) but not to tubulin (data not shown). The specificity of the goat anti-actin antibodies is shown in Figure 6, which shows a specific interaction with actin (Lane 1) but no cross-reaction with aldolase (Lane 2). This interaction between the filarial aldolase and actin might therefore provide a link between the two *Wolbachia* - *B. malayi* protein complexes we identified by LC-MS analysis and confirmed by other assays: *w*Bm0432 with *B. malayi* glycolytic enzymes and *w*Bm0152 with the *B. malayi* host cytoskeleton.

## Discussion

The filarial nematode and its endosymbiont are known to be co-dependent, but the cellular and molecular basis of this relationship has yet to be elucidated. Eliminating *Wolbachia* from the parasites using antibiotics affects molting, reproduction, and survival of the worms, indicating that the bacteria are crucial for the development of the parasite; thus, they represent an attractive target for control of the infections [20], [54], [55]. *Wolbachia* occupy the lateral cords of all stages of the filarial worms, and in female worms, they can be found in oocytes and embryonic stages within the uteri [56]. The *Wolbachia* OMPs, including the WSP-like family proteins were predicted to play an important role in communicating with the parasite to maintain homeostasis in the endosymbiotic relationship [36]–[39].

The *Wolbachia* surface protein *w*Bm0432 was found to associate with six enzymes involved in glycolysis: fructose-bisphosphate aldolase, triosephosphate isomerase, L-lactate dehydrogenase, enolase, glyceraldehyde-3-phosphate dehydrogenase (G3PD), and phosphoglycerate kinase. Notably, analysis of the available genome data revealed that *Wolbachia* lacks two glycolytic enzymes (6-phosphofructokinase and pyruvate kinase), and consequently its glycolytic pathway is thought to be defective and replaced by gluconeogenic enzymes [17], [18]. Accordingly, the energy source utilized by *Wolbachia* will depend on products produced by the *B. malayi* glycolytic pathway, such as pyruvate. The ability of *Wolbachia* to sequester several GEs onto their surface by creating a complex with *w*Bm0432 can increase the speed of glucose breakdown and thus synthesis of pyruvate. The pyruvate, once transported into the bacterial cell, can enter the TCA cycle, resulting in energy production [57].

The *Wolbachia w*Bm0152 protein was found to form a complex with the *B. malayi* cytoskeleton proteins actin and tubulin. This finding is concordant with many previous studies that have demonstrated a close association of *Wolbachia* and other intracellular bacteria with the host cell cytoskeleton. Both actin and tubulin are known to play an important role in distribution of intracellular organisms [58]–[63]. *Rickettsia*, obligate intracellular gram-negative bacteria and close relatives of *Wolbachia*, exhibit actin-based motility in the cytosol of host cells involving the RickA surface protein [60], [62]. *Listeria monocytogenes* and *Shigella flexneri* bacteria are internalized first into the host cells and then rapidly escape from the internalization vacuole into the cytosol, where they polymerize actin on their surface and initiate actin-based motility [64]. This property is not only restricted to *Rickettsia*, *Listeria* and *Shigella* but it also applies to other pathogens including apicomplexa and mycobacterial species such as *Mycobacterium marinum* and *Burkholderia pseudomallei* [59], [62]. The functional interactions between *Wolbachia* and the host microtubules have been well documented in arthropods where *Wolbachia* utilize microtubules for normal anterior localization in the *Drosophila* oocyte to ensure its transmission to the next generation [58]. Treatment with colchicine resulted in complete depolymerization of microtubules within the germ cells resulting in the failure of *Wolbachia* to localize to the anterior of the *Drosophila* oocyte. It was proposed that these interactions might also play a role in bacterial motility and replication, ultimately leading to their efficient maternal transmission [58]. However, the exact cellular and molecular mechanism underlying this association is still unknown.

In the present study of *B. malayi-w*Bm endosymbiotic relationship, we show that *w*Bm0152 forms a complex with both actin and tubulin but that it interacts directly only with actin based on the overlay assays. Accordingly, we hypothesize that *Wolbachia* interact with the host microtubules indirectly through the *w*Bm0152-actin link. In *B. malayi*, *Wolbachia* was previously shown to be present near the host's actin bundles and actin-rich rachis as determined by immunofluorescence [65]. It was shown that *Wolbachia* localize to the posterior of the egg upon fertilization and segregate asymmetrically during early embryogenesis in a lineage-specific manner. Therefore, it was speculated that these segregation patterns are responsible for determining the ultimate colonization of adult female tissues [65]. In this study we show that the WSP *w*Bm0152 protein co-localized with actin to the surface of the vacuole that surrounds *Wolbachia* by immunoelectron microscopy. Hence, the interaction between *w*Bm0152 and the host cytoskeleton might support *wBm* migration and segregation in host tissue during development, a process needed for its fitness and survival.

WSP *w*Bm0152 has been previously identified as a peptidoglycan associated lipoprotein (PAL), which is instrumental in the induction of innate toll receptor-mediated immune responses to *Wolbachia* that are associated with the pathogenesis of the human filarial parasites [66]. The diacyl lipid moieties present on native *w*Bm0152 have been shown to be important mediators in this response [66]. Thus, *w*Bm0152 is likely to form an important part of the peptidoglycan layer of the *Wolbachia* cell wall, and as such it is expected to be tightly embedded into the peptidoglycan matrix. If this is the case, *w*Bm0152 would become highly crosslinked in the experiments described above; perhaps explaining why no native peptides corresponding to *w*Bm0152 were detected in the analysis of the crosslinked products immunoaffinity purified using columns containing antibodies raised against recombinant *w*Bm0152.

Although the presence of *w*Bm0152 on the outer surface of *Wolbachia* is expected based upon its functional classification as a PAL, the present immunolocalization studies also suggest that it is present as well in the vacuole surrounding the endosymbiont. This finding is in keeping with previous studies that have shown the production of secretory vacuoles from *Wolbachia* [67] and the fact that *w*Bm0152 has previously been identified as a member of the secretome of the *Wolbachia* endosymbiont of *B. malayi* [40]. Together, these studies support the hypothesis that *w*Bm0152 might play an important role in the association of the endosymbiont to the cytoskeleton of the host cell.

Several reports have shown the dependency of nematode fitness on tubulin and actin functions. The targeting of β-tubulin in *B. malayi* adult worms and *Haemonchus contortus* larvae using the RNA interference (RNAi) technology led not only to a reduction in the levels of their transcripts but also to detrimental phenotypes [68], [69]. RNAi targeting of *B. malayi* β-tubulin resulted in parasite death [68] while in *H. contortus* it resulted in decreased L3 worm motility that slowed their development to L4, in comparison to control larvae [69]. RNAi targeting γ-tubulin in *B. malayi* resulted in cellular disorganization in embryos [70]. Similar effects were observed after knocking down transcript levels of actin (*Ls*-act) by RNAi in the rodent filaria *Litomosoides sigmodontis* [71]. Two phenotypes were seen with *Ls*-act targeted RNAi: paralysis, as demonstrated by the worm being stretched out and having slower movements and significant reduction in the release of microfilaria. It would be interesting to expand on these filarial RNAi studies and establish whether there is also a synergistic impact of the RNAi upon the biology of *Wolbachia* and its distribution within the filarial host.

In mammalian tissues, the enzymes of the glycolytic pathway utilize cytoskeleton as a matrix to keep phosphofructokinase, aldolase and G3PD in an optimal alignment for rapid substrate conversion [72], [73]. For instance, in red blood cells, several GEs (GAPDH, aldolase, and phosphofructokinase) assemble in complexes with the cell's cytoskeleton [72], [73], and their proximity with each other increases the speed of glucose breakdown. In previous studies of bovine brain tissue, aldolase, lactate dehydrogenase type-M, pyruvate kinase, and G3PD were shown to co-pellet with microtubules, with *K_d_* values between 1 and 4 µM [74]. More recent studies have shown that enolase isoforms purified from mouse brain and mouse striated muscles interact with microtubules during muscle satellite cell differentiation [75]. Aldolase in other systems is known to play a dual role, participating in glycolysis as soluble enzyme and forming a complex with the actin cytoskeleton filaments (F-actin) *in vitro* and *in vivo*, when it is enzymatically inactive [76], [77]. Aldolase is tetrameric and each monomer has the capacity to bind to F-actin [77]. In intracellular apicomplexan parasites, the translocation of parasites is facilitated by a link between cell surface adhesins, aldolase and actin where aldolase is a bridge between the adhesins and the cytoskeleton [51].

**Figure 7:**
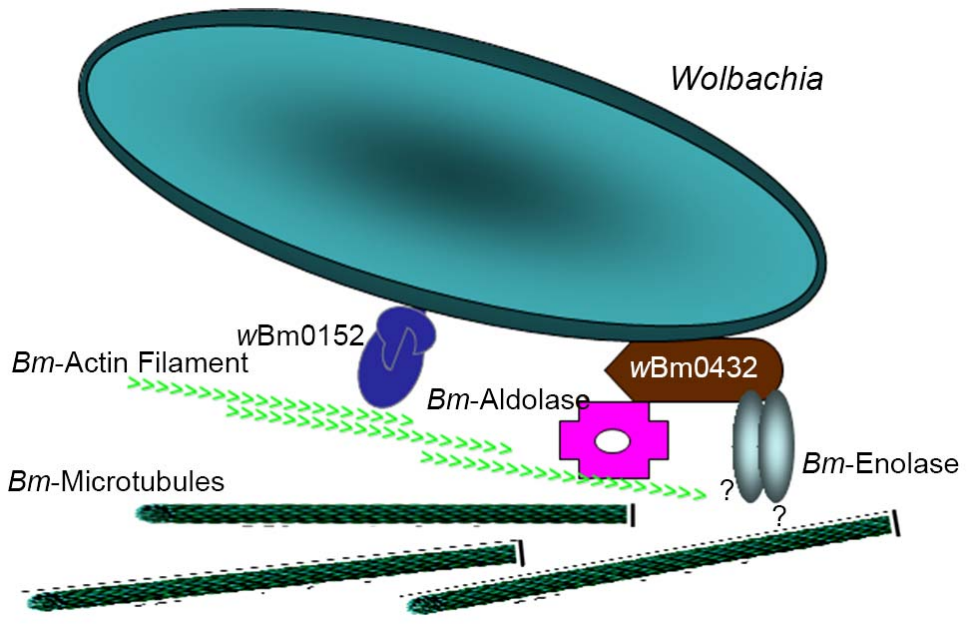
The model for *Bm*-*w*Bm interactions. The complex of *w*Bm0432 with the GE proteins is potentially associated with the complex of *w*Bm0152/cytoskeletal proteins via aldolase and/or enolase.

The data we present suggest that *B. malayi* aldolase might provide a link between the two protein complexes we identified in this study. *Bm*-aldolase binds to actin, while *w*Bm0432 binds strongly to aldolase and possibly also to enolase and to some extent to G3PD and triosephosphate isomerase, but not to actin or tubulin. We therefore hypothesize that as in the apicomplexa, malaria and *Toxoplasma* [78], [79], aldolase might play a dual role also in the *B. malayi-Wolbachia* endosymbiotic relationship. In addition to its central role in glycolysis, aldolase might also mediate the interaction between *Wolbachia* and the host's cytoskeleton (Fig. 7). First, it may complex with the WSP *w*Bm0432 protein and other GEs, providing *Wolbachia* with sequestered production of pyruvate and thus ATP. This would be in keeping with the “supplemental mitochondrion” hypothesis, which has been proposed as one role that the *Wolbachia* endosymbiont might play in the host-endosymbiont relationship [80]. It may also function as an anchor between *Wolbachia* and the *B. malayi* cytoskeleton using the ATP produced at the surface as an energy source to engage the actin cytoskeletal network to support its motility and distribution within the host.

Future studies will verify the functional involvement of the *w*Bm0432/glycolytic enzymes and *w*Bm0152/cytoskeletal proteins in *Wolbachia's* transmission patterns within the *B. malayi* host. Additional studies will also be needed to validate the essential role of these two *Bm-w*Bm interactomes for the survival of *B. malayi* and its co-dependency on *Wolbachia*.

## Supporting Information

Figure S1 **Fixation artifact observed in the vacuole surrounding** ***Wolbachia*** **.** Images of *Wolbachia* residing in the hypodermal chord of *B. malayi* using two different fixation methods are presented in Panels A–D. Panels A and B are representative of LR white embedded specimens exhibiting the typical “halo” surrounding *Wolbachia* in the hypodermal chord tissue. Panels C and D show the same cross-section sample that was prepared utilizing the fixation protocol for Epon embedding, and which lacks the large halos surrounding the *Wolbachia*.
(TIF)

Figure S2 **Structure of the host's vacuole surrounding** ***Wolbachia*** **in an LR white preparation.** A higher magnification of the sample shown in Figure S1, Panels A and B is showing a single *Wolbachia* with a surrounding vacuole exhibiting the halo artifact. The cell wall of *Wolbachia* is clearly shown (black arrow), as is a portion of the vacuole boundary (black arrowheads). In addition, a filamentous network is evident along the vacuole border (thick white arrows).
(TIF)
