# Expression of Concern: A Potential Role for the Interaction of *Wolbachia* Surface Proteins with the *Brugia malayi* Glycolytic Enzymes and Cytoskeleton in Maintenance of Endosymbiosis

**DOI:** 10.1371/journal.pntd.0011860

**Published:** 2023-12-29

**Authors:** The PLOS Neglected Tropical Diseases Editors

After this article [1] was published, concerns were raised about Fig 4.

Specifically:

- In Fig 4A, when levels are adjusted to visualize the background there appear to be similar repeating patterns present in the background in lanes 2 and 4 despite these lanes representing different experimental conditions.

The corresponding author of this article stated that the underlying data for this article was no longer available and had been deleted in accordance with institutional data retention guidelines. In the absence of the data underlying this panel the above concern cannot be resolved.

In light of this issue, the *PLOS Neglected Tropical Diseases* Editors issue this Expression of Concern.
