# Supplementary figures and images for: A Potential Role for the Interaction of Wolbachia Surface Proteins with the Brugia malayi Glycolytic Enzymes and Cytoskeleton in Maintenance of Endosymbiosis
Source: PLoS Negl Trop Dis. 2013 Apr 4;7(4):e2151. doi: 10.1371/journal.pntd.0002151 (PMC3617236; doi:10.1371/journal.pntd.0002151)

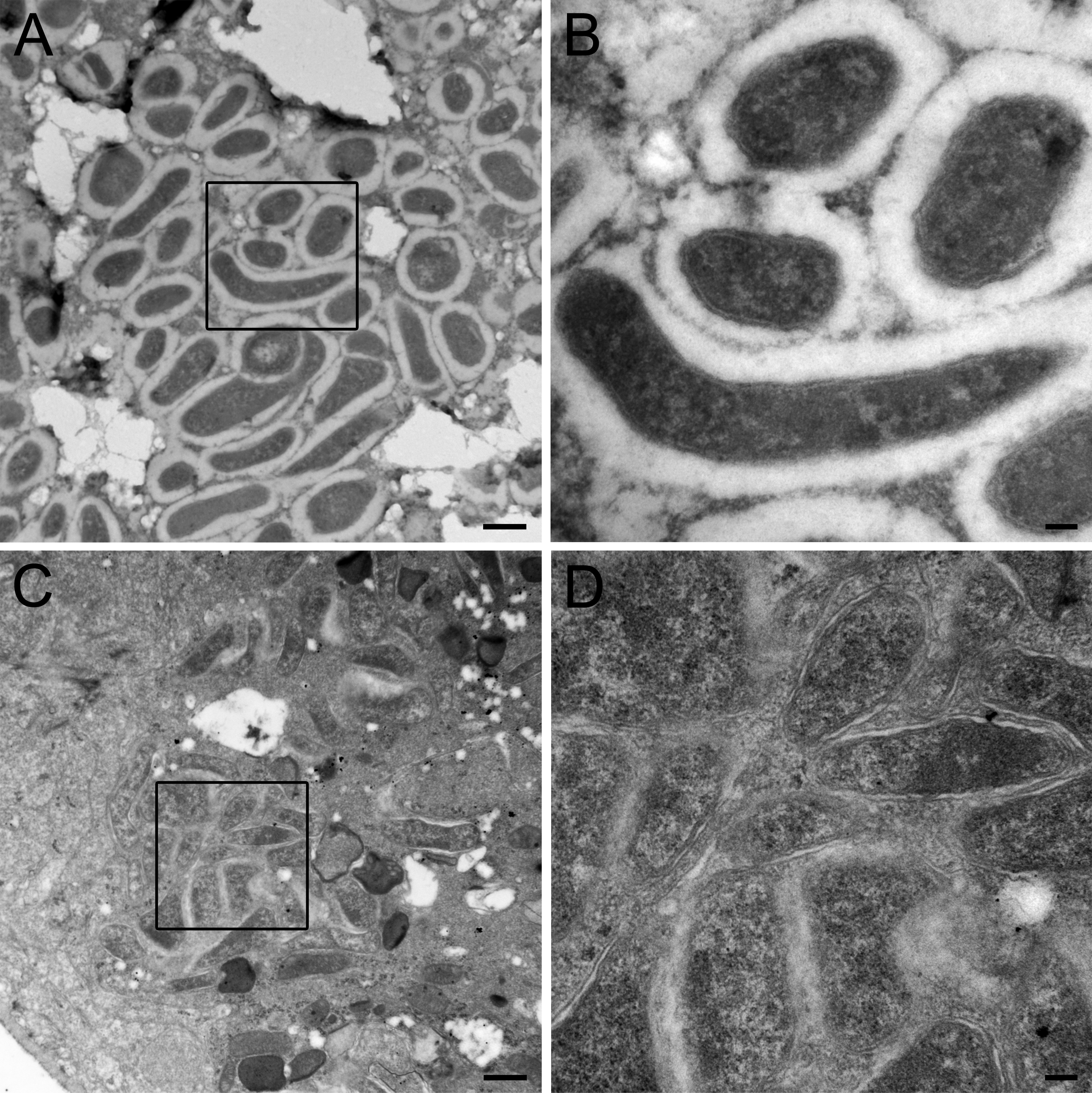

Supplement: Figure S1 — Fixation artifact observed in the vacuole surrounding Wolbachia . Images of Wolbachia residing in the hypodermal chord of B. malayi using two different fixation methods are presented in Panels A–D. Panels A and B are representative of LR white embedded specimens exhibiting the typical “halo” surrounding Wolbachia in the hypodermal chord tissue. Panels C and D show the same cross-section sample that was prepared utilizing the fixation protocol for Epon embedding, and which lacks the large halos surrounding the Wolbachia. (TIF) [file pntd.0002151.s001.tif]

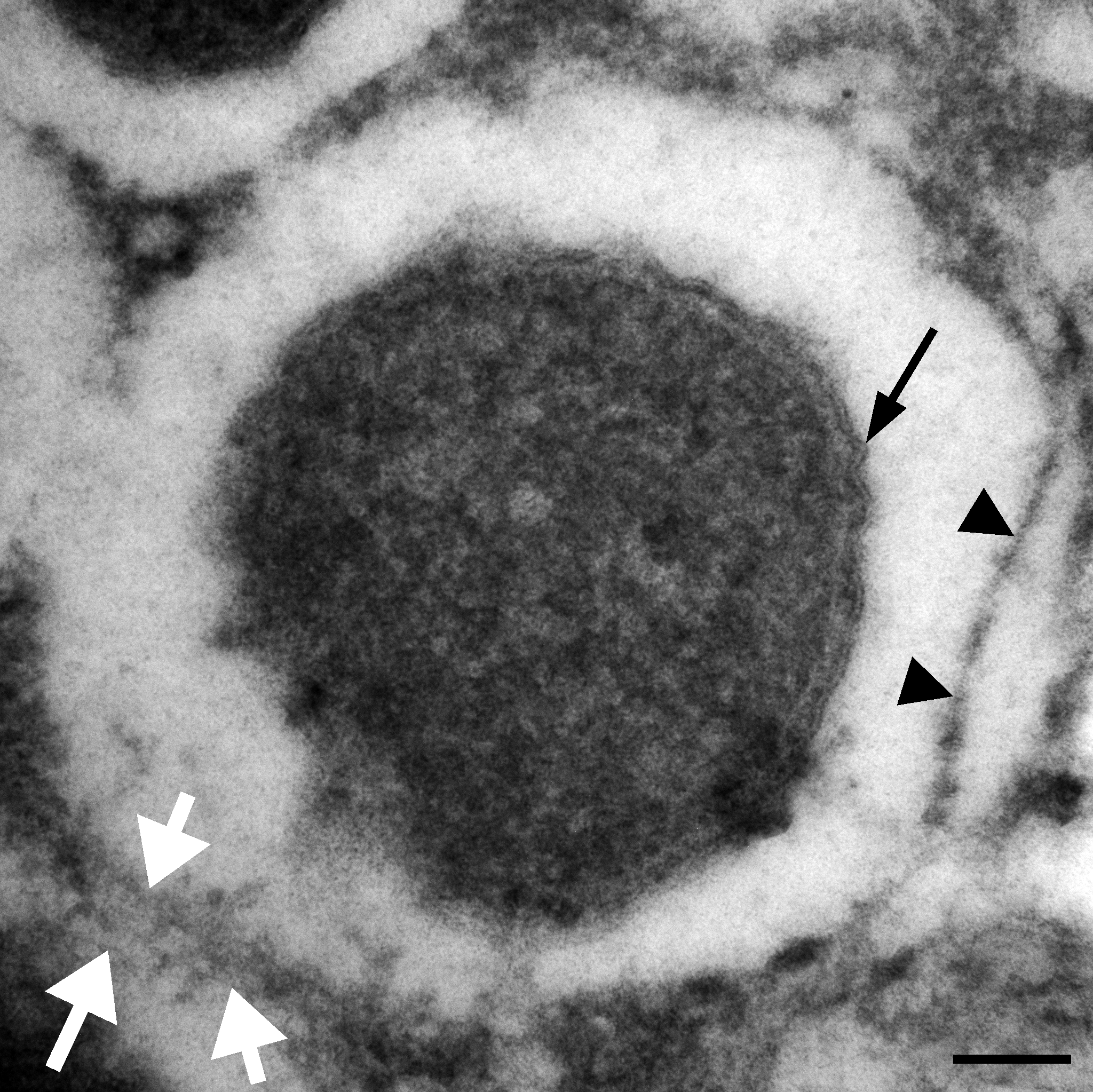

Supplement: Figure S2 — Structure of the host's vacuole surrounding Wolbachia in an LR white preparation. A higher magnification of the sample shown in Figure S1, Panels A and B is showing a single Wolbachia with a surrounding vacuole exhibiting the halo artifact. The cell wall of Wolbachia is clearly shown (black arrow), as is a portion of the vacuole boundary (black arrowheads). In addition, a filamentous network is evident along the vacuole border (thick white arrows). (TIF) [file pntd.0002151.s002.tif]
